# A novel mechano-enzymatic cleavage mechanism underlies transthyretin amyloidogenesis

**DOI:** 10.15252/emmm.201505357

**Published:** 2015-08-18

**Authors:** Julien Marcoux, P Patrizia Mangione, Riccardo Porcari, Matteo T Degiacomi, Guglielmo Verona, Graham W Taylor, Sofia Giorgetti, Sara Raimondi, Sarah Sanglier-Cianférani, Justin LP Benesch, Ciro Cecconi, Mohsin M Naqvi, Julian D Gillmore, Philip N Hawkins, Monica Stoppini, Carol V Robinson, Mark B Pepys, Vittorio Bellotti

**Affiliations:** 1 Department of Chemistry, University of OxfordOxford, UK; 2 Laboratoire de Spectrométrie de Masse BioOrganique (LSMBO), University of Strasbourg UDSStrasbourg, France; 3 Wolfson Drug Discovery Unit, Centre for Amyloidosis and Acute Phase Proteins, Division of Medicine, University College LondonLondon, UK; 4 Department of Molecular Medicine, Institute of Biochemistry, University of PaviaPavia, Italy; 5 Institute of Nanoscience S3, Consiglio Nazionale delle RicercheModena, Italy; 6 Department of Physics, Informatics and Mathematics, University of Modena and Reggio EmiliaModena, Italy

**Keywords:** amyloid, mechano-enzymatic cleavage, transthyretin

## Abstract

The mechanisms underlying transthyretin-related amyloidosis *in vivo* remain unclear. The abundance of the 49–127 transthyretin fragment in *ex vivo* deposits suggests that a proteolytic cleavage has a crucial role in destabilizing the tetramer and releasing the highly amyloidogenic 49–127 truncated protomer. Here, we investigate the mechanism of cleavage and release of the 49–127 fragment from the prototypic S52P variant, and we show that the proteolysis/fibrillogenesis pathway is common to several amyloidogenic variants of transthyretin and requires the action of biomechanical forces provided by the shear stress of physiological fluid flow. Crucially, the non-amyloidogenic and protective T119M variant is neither cleaved nor generates fibrils under these conditions. We propose that a mechano-enzymatic mechanism mediates transthyretin amyloid fibrillogenesis *in vivo*. This may be particularly important in the heart where shear stress is greatest; indeed, the 49–127 transthyretin fragment is particularly abundant in cardiac amyloid. Finally, we show that existing transthyretin stabilizers, including tafamidis, inhibit proteolysis-mediated transthyretin fibrillogenesis with different efficiency in different variants; however, inhibition is complete only when both binding sites are occupied.

## Introduction

The determinants of induction, anatomical distribution and rates of progression of clinical transthyretin (TTR) amyloid deposition are poorly understood. High-resolution 3D crystallographic structures of native wild-type TTR and several amyloidogenic variants have identified no relevant features (Palaninathan, 2012), and the pathogenic mechanism is probably hidden within the dynamics of folding and subunit assembly. The native tetrameric assemblies of all amyloidogenic TTR variants are less stable *in vitro* than those of wild-type TTR, and the dissociated protomers have a strong propensity to self-aggregate (Merlini & Bellotti, 2003), but there is no direct evidence that this fully explains fibrillogenesis *in vivo*.

A key role for partial proteolysis in the initiation of amyloid fibril formation in general has been debated extensively (Westermark *et al*, 1996). The residue 49–127 C-terminal fragment is a major component of *ex vivo* TTR amyloid fibrils (Thylen *et al*, 1993; Ihse *et al*, 2013), regardless of the presence, nature or position of any amyloidogenic mutation (Bergstrom *et al*, 2005). In particular, the detection of this fragment in tissue biopsies correlates with amyloid deposition in the heart (Ihse *et al*, 2013) and poor clinical prognosis (Gustafsson *et al*, 2012).

The extremely rare S52P TTR variant is the least stable TTR variant and causes the most aggressive phenotype of hereditary systemic TTR amyloidosis (Mangione *et al*, 2014). We have recently shown that limited proteolysis of S52P TTR generates the 49–127 fragment that, if released under physiological fluid agitation, rapidly self-aggregates into very stable aggregates (Mangione *et al*, 2014) together with the full-length protein. The molecular mechanism of this remarkable phenomenon and whether it underlies amyloid fibrillogenesis by other amyloidogenic TTR variants, including the wild-type protein, are fundamental to understanding the pathogenesis of this amyloid disease.

## Results

### Release of proteolytic fragments from the tetramer

**Figure 1:**
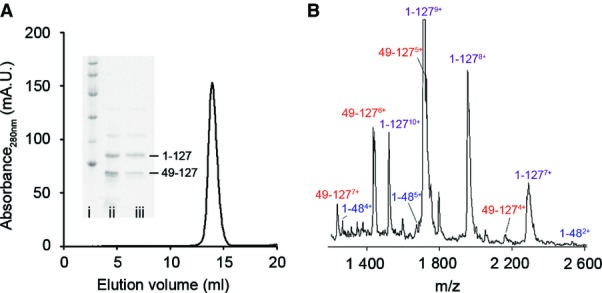
Fragments are retained within the tetramer after limited proteolysis Size-exclusion chromatography elution profile of S52P TTR after limited proteolysis shows a single peak eluting at the same retention volume (14.3 ml) as TTR tetramer. Inset, SDS–15% PAGE analysis confirms that the tetramer eluting in the peak is partially cleaved. Lane i: marker proteins (14.4, 20.1, 30.0, 45.0, 66.0 and 97.0 kDa); lane ii: S52P TTR after limited proteolysis; lane iii: SEC eluted peak. Arrows show the full-length protomer (residues 1–127) and the tryptic 49–127 fragment.
Mass spectrum of SEC eluted peak of trypsin-digested S52P TTR. The sample was partially denatured by twofold dilution in 25% acetonitrile and 0.05% formic acid, showing the presence of full-length protomer (purple) and both 1–48 (blue) and 49–127 (red) fragments.

**Figure 2:**
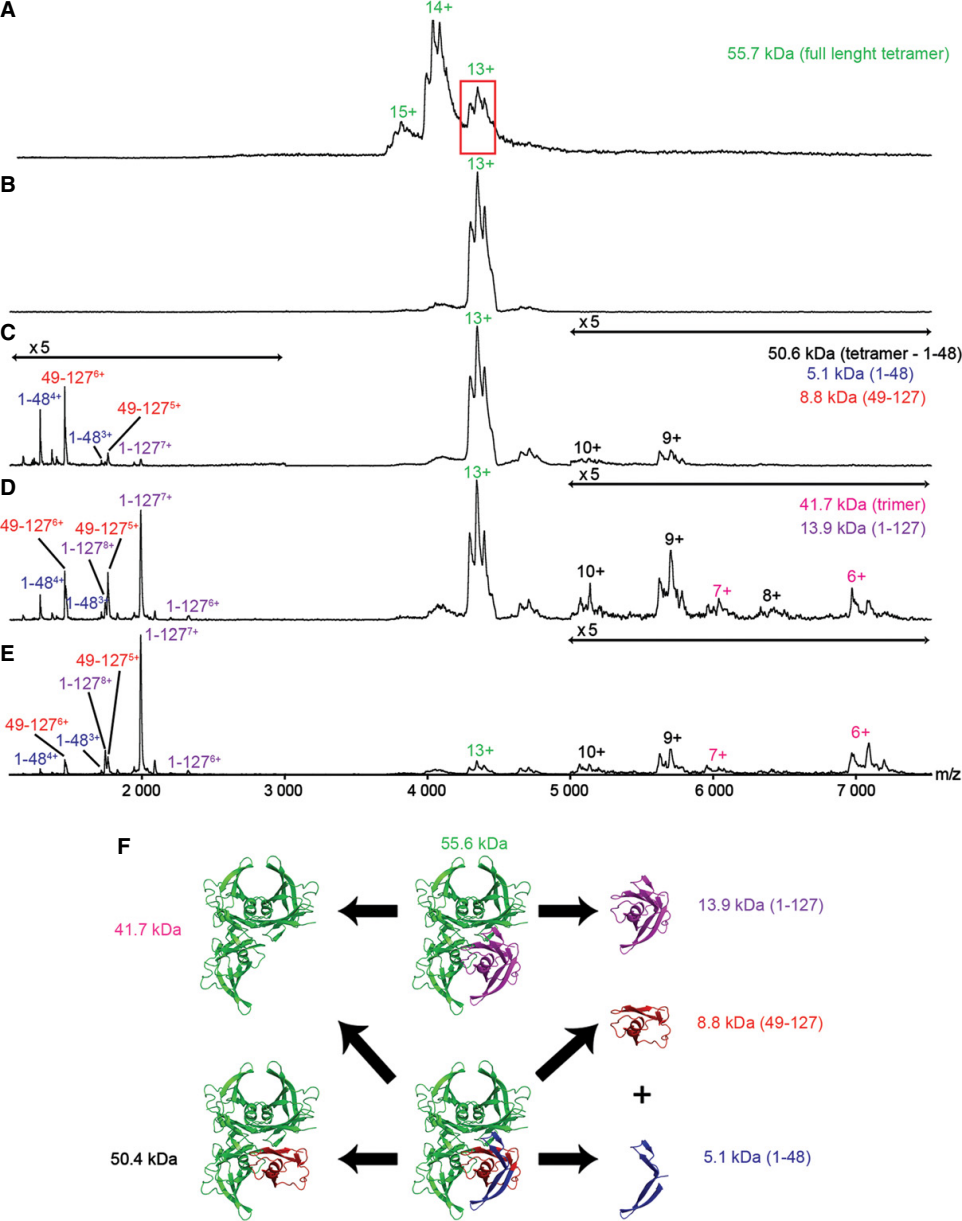
Following limited proteolysis, truncated fragments and full-length protomer are released from the tetramer at increasing energy A Mass spectrum of native S52P TTR (green) obtained at low collision energy. The red box highlights the 13^+^ charge state precursor.
B The 13^+^ charge state was then selected and subjected to increasing collision energy.
C–E The cleaved fragments 1–48 (blue) and 49–127 (red) were released from the tetramer before dissociation of the full-length protomer (purple). The adducts visible on the tetramer were identified as the tryptic peptide CPLMVK (Appendix Figs S6 and S7). Double arrows indicate the *m/z* ranges where signal intensity was magnified fivefold.
F Scheme showing the release of cleaved fragments and full-length protomer obtained by collision-induced dissociation (CID) after limited proteolysis. The experimental molecular masses of the different complexes in (A–E) are in good agreement with the theoretical values in (F); the small difference(s) arise from incomplete desolvation and the inherent measurement error of the method.

Trypsin digestion of S52P TTR under physiological conditions (PBS, pH 7.4, 37°C) without fluid agitation cleaves some but not all protomers (Mangione *et al*, 2014) at residue Lys48. Under the mild shear force conditions of low-pressure size-exclusion chromatography (SEC), the cleaved protein eluted as a single peak with the same V_e_ as native undigested tetrameric TTR (Fig 1A), and with both full-length protomer and the truncated residue 49–127 fragment detected by SDS–PAGE of the chromatographic peak (Fig 1A, inset). The residue 1–48 TTR, which cannot be readily detected in our electrophoretic system, was confirmed by non-native MS of the SEC fraction (Fig 1B). The cleaved protein thus remained exclusively in the native tetrameric assembly. Similarly in non-denaturing MS (native MS) with low acceleration voltage, only complete native mass tetramers were observed in the trypsin-digested protein. Despite the expectation that both intact tetramers and tetramers with at least one cleaved protomer would be present, there was no evidence for either truncated tetramers, having lost fragments 1–48 or 49–127, or the fragments themselves (Fig 2A).

To release the peptide in the gas phase, we mass-selected the 13^+^ charge state of the tetramer(s) and increased the acceleration voltage. Both the 1–48 and 49–127 fragments were visible in the low *m/z* region of the resulting mass spectrum, together with a stripped trimer at high *m/z*. With further increases in accelerating voltage, the intact tetramers released full-length protomers which progressively overwhelmed the signal arising from the fragments (Fig 2A–E). The generation of a truncated tetramer at higher energy, containing three intact protomers and the fragment 49–127, strongly suggests that the fragment 1–48 is the first to be released (Fig 2F). The release of these fragments, at lower energy than the full-length protomers, indicates that the cleavage has destabilized the truncated protomer.

### Single proteolytic cleavage destabilizes the tetramer

We compared the stability of the different full-length protomers and/or proteolytic fragments as described before (Keetch *et al*, 2005; Hyung *et al*, 2010), by selecting charge states and activation in the gas phase. We observed that after proteolysis of S52P TTR, the tetramers were less stable (Appendix Fig S1A, blue versus red line). Moreover, the fragments were released at lower acceleration voltages than the full-length subunits (Appendix Fig S1B, dotted versus dashed lines). The difference observed here would be even more dramatic if each tetramer contained at least one cleaved protomer. As expected, the non-cleaved subunits showed similar dissociation patterns, irrespective of whether or not the protein had been incubated with trypsin (Appendix Fig S1B, blue and red dashed lines). A similar experiment was performed on wild-type TTR, which is resistant to limited proteolysis in the absence of fluid stirring (Mangione *et al*, 2014). The dissociation pattern (Appendix Fig S1, green lines) was similar to that of the non-cleaved S52P variant TTR indicating a similar overall stability of the two tetramers.

### Molecular dynamics simulation

**Figure 3:**
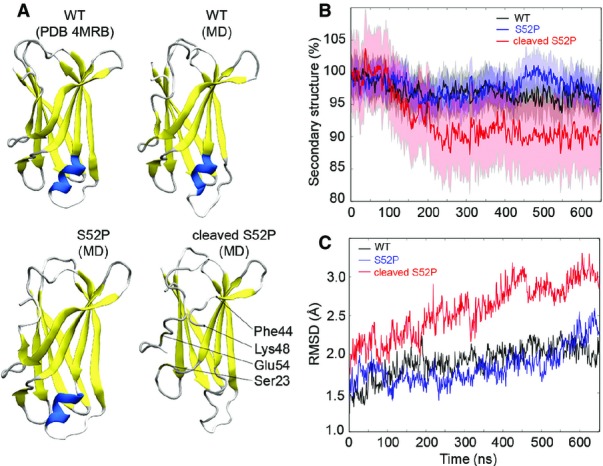
Cleavage at residue Lys48 induces an allosteric conformational change Structures of wild-type and S52P subunits after 500-ns MD simulation are similar to the original wild-type crystal structure (Mangione *et al*, 2014). Conversely, the cleavage of S52P TTR induces an unfolding in the 43–48 strand, followed by loss of secondary structure in the Ser23, Glu54 regions and the EF-helix (in blue), respectively.
Relative percentage of secondary structure retained during the simulations of wild-type (black) and of the S52P variant TTR with (red) and without (blue) cleavage. The solid lines show average values measured in four independent subunits. The full colour areas represent standard deviations.
Root mean square deviations during the simulations of wild-type (black) and S52P variant TTR before (blue) and after (red) cleavage.

In order to study the effect of proteolytic cleavage, we undertook molecular dynamics (MD) simulations for wild-type TTR and the S52P variant, both with and without cleavage at residue Lys48. As residue 48 is located far from the two different dimer interfaces, the four subunits were considered as independent and cleavage was performed on each protomer, in order to increase sampling. Simulations were optimized to reproduce physiological conditions as far as possible (see Materials and Methods). During the first 500 ns, wild-type and S52P TTR maintained folded structures closely resembling their native crystal structure (Mangione *et al*, 2014) with no apparent unfolding (Fig 3A). Following cleavage at Lys48, MD simulations of S52P TTR revealed a dramatic unfolding of the 43–48 strand accompanied by the allosteric loss of the EF-helix (Fig 3B and C; Movie EV1). This observation enables us to rationalize our MS results, in which the 1–48 fragment was released from the cleaved tetramer prior to the 49–127 polypeptide, since MD simulation shows that the latter remains engaged in inter-subunit contacts.

### The effect of cleavage on hybrid tetramers of wild-type/S52P TTR

**Figure 4:**
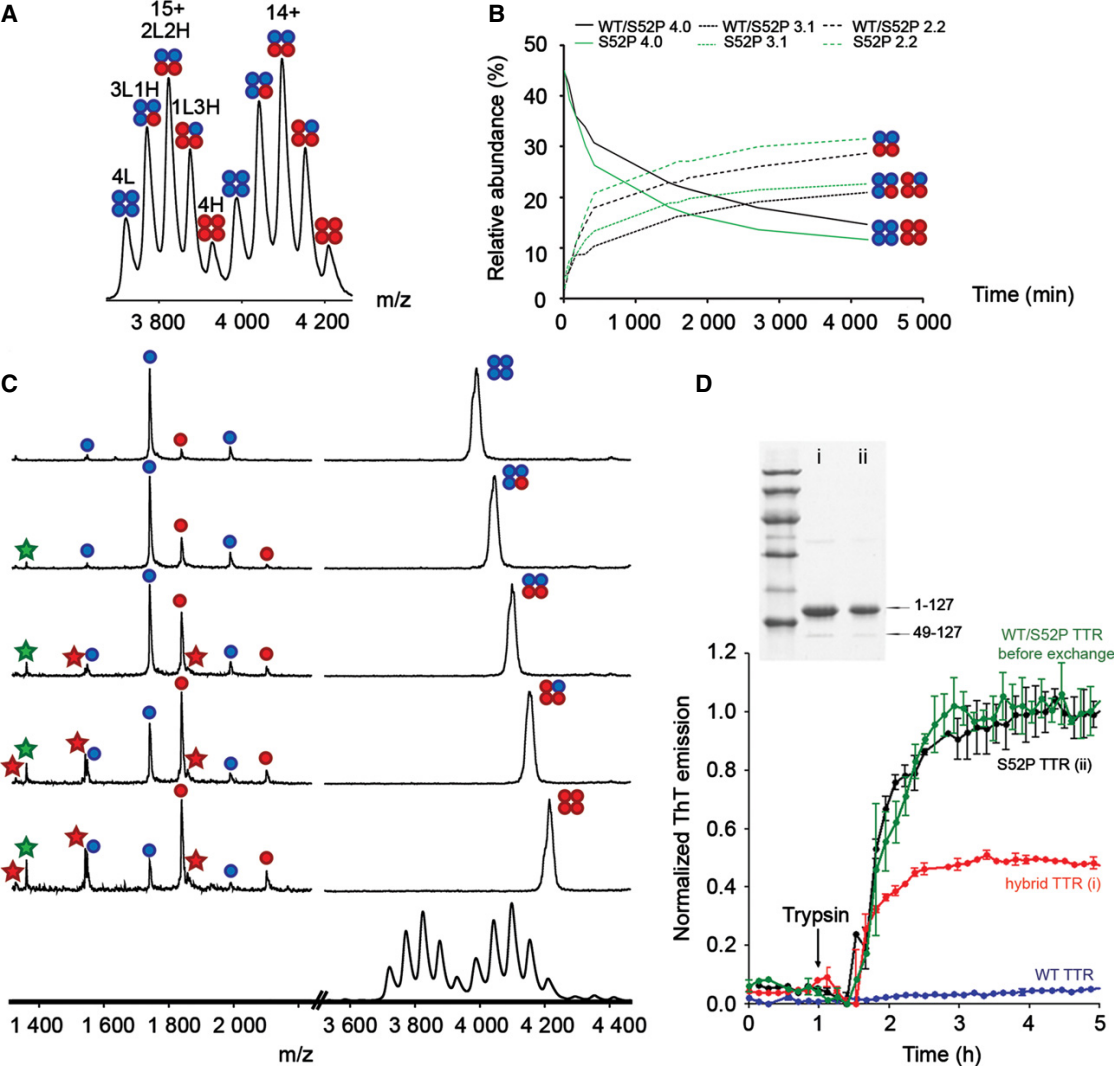
S52P/wild-type hybrid TTR: formation, proteolysis, fragment release and fibril formation Subunit exchange experiments between light recombinant wild-type (L, blue) and heavy (^13^C^15^N) S52P TTR (H, red) showing that both proteins can exchange subunits.
Comparison of exchange between light recombinant wild-type/heavy S52P TTR (black lines) and light S52P/heavy S52P TTR (green lines) shows that dynamics are faster when only S52P TTR is present. Light subunit, blue; heavy subunit, red.
Subunit exchange between light wild-type (blue) and heavy S52P TTR (red) followed by limited proteolysis. All species were isolated and dissociated (MSMS) to yield 1–48 and 49–127 fragments (green and red stars, respectively) that can be detected from any of the intermediate species containing at least one monomer of heavy S52P TTR. The presence of traces of S52P TTR in the wild-type TTR spectrum (top) and vice versa (bottom) is due to partial overlap of the different molecular ion species during selection. The lowest spectrum corresponds to the full-scan MS spectrum showing all the species.
Normalized thioflavin T (ThT) fluorescence data of homotetrameric S52P (9 μM, black) and homotetrameric wild-type TTR (9 μM, blue); hybrid TTR (wild-type TTR, 9 μM + S52P TTR, 9 μM) before (green) and after (red) 72-h subunit exchange, respectively. Data plotted as mean ± SD of at least three replicates. Inset, SDS–15% PAGE under reducing conditions of wild-type/S52P hybrid TTR (i) and S52P TTR (ii) after 5-h incubation with trypsin at 37°C. Marker proteins (14.4, 20.1, 30.0, 45.0, 66.0 and 97.0 kDa) are included. Arrows indicate the full-length protomer (residues 1–127) and the tryptic 49–127 fragment, respectively.

The exchange of protomers between tetramers of TTR has been described previously (Keetch *et al*, 2005; Hyung *et al*, 2010), and certain point mutations known to destabilize the tetramer (e.g. L55P TTR) have been shown to enhance the rate of subunit exchange between tetramers (Keetch *et al*, 2005). We reasoned that increased subunit exchange rates could also occur in S52P TTR following trypsin cleavage, given the reduced stability of the tetramer. To explore this hypothesis, we first incubated wild-type TTR with isotopically labelled (^13^C^15^N) S52P TTR for 72 h. The resulting mass spectrum revealed different populations of tetramers containing the following: 4L, 3L1H, 2L2H, 1L3H and 4H subunits corresponding to L: light wild-type TTR, and H: heavy S52P TTR (Fig 4A). After 3 days, subunit exchange reached equilibrium with statistical distribution of subunits among the heterotetramers confirming that subunit exchange was complete.

We followed the kinetics of subunit exchange between wild-type TTR and the S52P variant and plotted the relative abundance of each tetrameric population with time (Fig 4B, black lines). Similarly, in a second experiment we monitored the kinetics of exchange of S52P TTR protomers alone using labelled and unlabelled protein (Fig 4B, green lines). We then compared the exchange kinetics of wild-type and (^13^C^15^N) S52P TTR with that of labelled and unlabelled S52P TTR. We found that subunit exchange was notably faster for S52P TTR alone suggesting a more dynamic state for pure S52P TTR than for the S52P/wild-type hybrid TTR.

In a third experiment, we performed limited proteolysis with trypsin on wild-type and labelled S52P TTR protomers which had pre-equilibrated for 3 days to form hybrid tetramers (Fig 4C). The mass spectrum recorded for the proteolysed tetramers (Fig 4C*,* lower) was indistinguishable from that of the fully exchanged non-proteolysed tetramers (Fig 4A). By performing tandem MS, we were able to isolate and activate each of the different tetramers. This enabled us to release the proteolytic fragment if formed. Activation of the isolated 4L species (Fig 4C, top) confirmed that it contained only full-length wild-type subunits. Similarly, the 4H species was found to contain a mixture of both protomers and fragments of S52P TTR. Interestingly, we were able to release fragments from all hybrid tetramers containing at least one S52P TTR protomer. Moreover, the intensity of the fragments increased with the number of S52P TTR subunits within the tetramer. Under these proteolysis conditions, in which the amount of variant protomers was kept identical in both hybrid and homotetrameric S52P TTR (Fig 4D), wild-type subunits were not cleaved, confirming the resistance to trypsin digestion of wild-type TTR in this state (see Materials and Methods) and demonstrating that S52P variant TTR is more susceptible to proteolysis.

### The effect of increased shear stress on the cleavage of TTR variants

**Figure 5:**
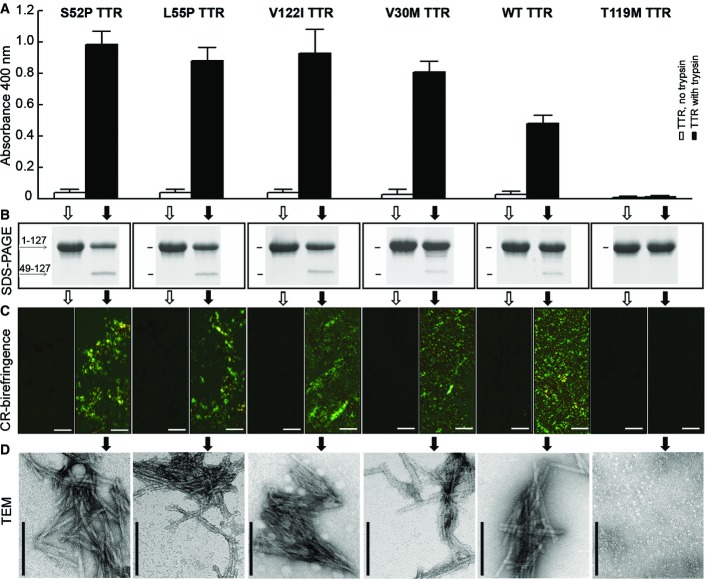
Effect of trypsin on amyloidogenesis of S52P, L55P, V122I, V30M, WT and T119M variant TTR Aggregation was quantified as spectrophotometric turbidity at 400 nm of samples incubated in PBS pH 7.4 at 37°C for 72 h with magnetic bar agitation in the absence (open bars) or presence (solid bars) of trypsin at an enzyme:substrate ratio of 1:200. Each bar shows mean ± SD of three replicates.
Selective proteolytic cleavage of TTR was monitored by SDS–PAGE under reducing conditions, stained with Coomassie Blue. Quantification of each electrophoretic band is shown in Appendix Fig S3.
Amyloid was identified by light microscopy of Congo red-stained specimens viewed under crossed polarized light (scale bar, 10 μM).
Negatively stained transmission electron microscopy (TEM) (scale bar, 100 nm) of TTR samples treated with trypsin as described above.

Based on the 3D structure of S52P TTR (PDB 4MRC), we hypothesized that its susceptibility to proteolytic cleavage depends on the high flexibility of the single peptide bond within the loop between strands C-D in the variant (Mangione *et al*, 2014). It is possible that fluid stirring could expose TTR to forces that enhance the flexibility of the C-D loop. We investigated this idea with five naturally occurring disease-associated variants and monitored aggregation (Fig 5A) in the presence of trypsin under fluid stirring and the larger air/water interface of 1.5 cm^2^ of a glass vial rather than 0.2 cm^2^ of a microtitre well. We found that, in these conditions, wild-type and four of the variants became susceptible to trypsin cleavage. SDS–PAGE confirmed that the 49–127 C-terminal fragment was released from the proteolysed tetramer in each case (Fig 5B). We then tested the solutions for the presence of fibrils and found that, after 72 h, genuine amyloid fibrils had formed when proteolysis had occurred (Fig 5). The pathognomonic amyloid apple-green birefringence under polarized light after Congo red staining (Fig 5C) and the typical fibrillar structure (Fig 5D) were observed in all those samples. A sixth variant, the non-amyloidogenic T119M TTR, was resistant to proteolysis and did not form fibrils.

SDS–PAGE analysis of the fibrillar material confirmed that, in all the amyloidogenic variants and the wild-type, the main component was the residue 49–127 polypeptide together with a minor band of uncleaved monomer (Appendix Fig S2). The increase in the concentration of the fragment in solution and the consumption of the protomer were not correlated, probably because of a combination of two different influences on the TTR fragment once it was released from the tetramer. The truncated polypeptide is most likely largely unfolded and can undergo two competing reactions: either being further digested by the protease or accumulated into fibrillar aggregates. Susceptibility to the proteolytic cleavage was therefore more reliably monitored by consumption of the intact protomer than direct estimation of the cleavage product in the trypsin-treated samples as shown in Figure 5B. S52P TTR was substantially more susceptible to proteolytic cleavage than wild-type TTR, and only T119M TTR was totally resistant to digestion (Appendix Fig S3A). Under the effect of agitation alone and in the absence of trypsin, none of the proteins were cleaved and maintained their solubility (Appendix Fig S3B).

### TTR ligands can prevent mechano-enzymatic cleavage

We previously reported that thyroxine binding by S52P TTR does not prevent trypsin cleavage of this variant and its consequent fibril formation, suggesting that small ligands may have only marginal effects on this proteolysis-primed pathway of TTR amyloidogenesis (Mangione *et al*, 2014). Here, we investigated the effect of two small stabilizing ligands, tafamidis (Coelho *et al*, 2013) and diflunisal (Berk *et al*, 2013), which are currently being trialled for the treatment of TTR amyloidosis. Protein/ligand complexes were subjected to our established fibrillogenesis assay in which the thioflavin T signal is monitored before and after the addition of trypsin with agitation and compared with the aggregation of the protein alone (see Materials and Methods). Consistent with the lack of efficacy of thyroxine, neither tafamidis nor diflunisal, even in large molar excess over TTR, inhibited the proteolytically mediated fibrillogenesis of S52P TTR (Appendix Fig S4).

**Figure 6:**
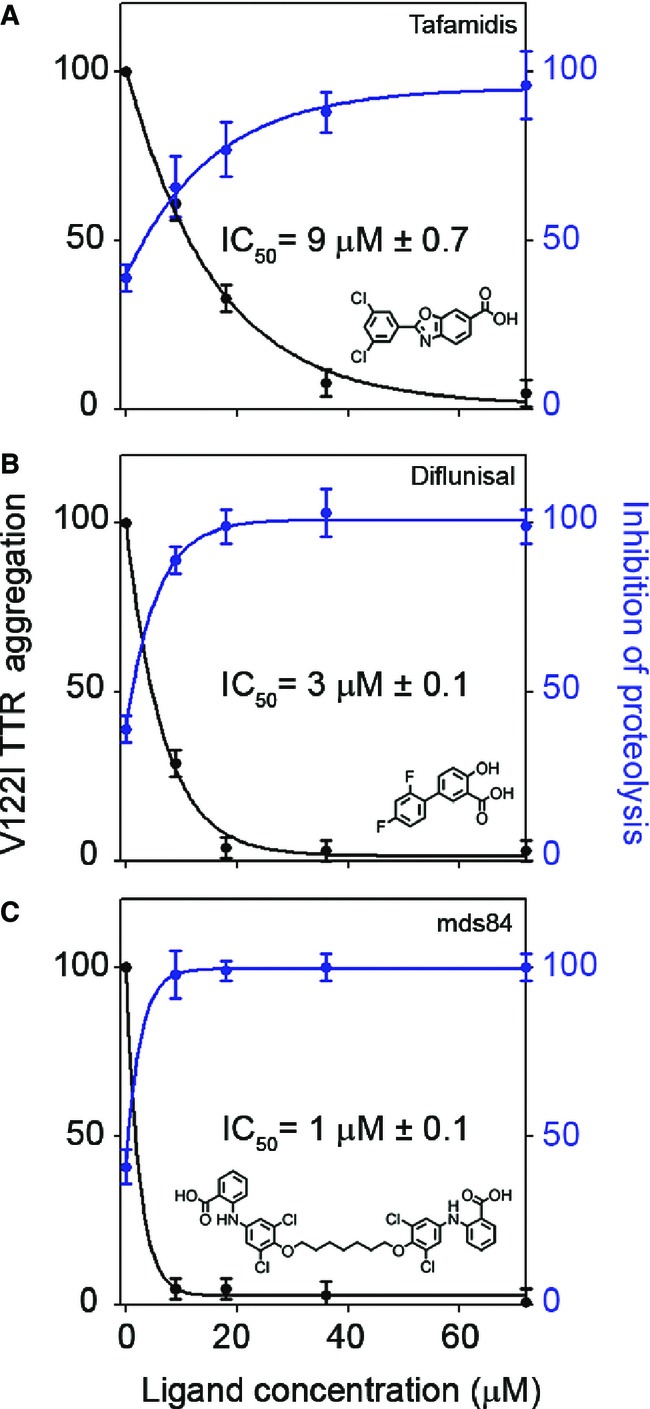
Proteolysis and fibrillogenesis of V122I TTR in the presence of TTR stabilizing drugs A–C Aggregation of 18 μM V122I TTR was monitored by turbidity at 400 nm after addition of trypsin in the presence of 0, 9, 18, 36 and 72 μM of (A) tafamidis, (B) diflunisal and (C) mds84, respectively, for 96 h; aliquots of each sample were analysed by SDS–15% PAGE under reducing conditions. Values of turbidity (black line) at 400 nm were normalized to 100% for aggregation of the protein alone. Intensities of the SDS–PAGE band corresponding to the intact protomer in the whole mixture (blue line) were normalized to 100% for the same band of the protein before addition of trypsin. The solid lines represent the nonlinear fit to the experimental data. IC_50_ values for TTR aggregation curves and corresponding ligand structures are included. All data shown represent mean ± SD of three independent experiments.

Since susceptibility to proteolysis correlates with protein stability, it is possible that cleavage and dissociation of the very unstable S52P TTR variant was too rapid to be inhibited by TTR ligands. In contrast, V122I TTR, the most prevalent TTR variant causing cardiac amyloidosis and with its clinical role currently under extensive investigation (Quarta *et al*, 2015), has stability and susceptibility to cleavage intermediate between S52P and wild-type TTR. We therefore monitored the effects of two monovalent TTR stabilizers, tafamidis (Bulawa *et al*, 2012) and diflunisal (Berk *et al*, 2013), and the experimental palindromic compound mds84 (Kolstoe *et al*, 2010), on fibrillogenesis of V122I under conditions suitable for enzymatic cleavage and formation of fibrils. At 96 h, when fibrils were formed in the absence of any ligand, we measured the intensities of the full-length protomer electrophoretic bands and the turbidity at 400 nm in the presence and absence of the ligands. The different ligands inhibited both proteolysis and aggregation with varying levels of potency (Fig 6), the palindromic mds84 (Kolstoe *et al*, 2010) being the most effective, followed by diflunisal and tafamidis (Berk *et al*, 2013; Coelho *et al*, 2013).

## Discussion

S52P TTR variant provides a unique model for elucidation of the pathway of TTR amyloidogenesis primed by proteolytic cleavage of the 48–49 peptide bond. In circulating plasma of carriers heterozygous for this TTR mutation, wild-type/S52P TTR hybrid tetramers will be present (Schneider *et al*, 2001). We therefore analysed both homotetrameric S52P TTR and hybrid tetramers and observed selective proteolytic cleavage of S52P TTR followed by release of truncated polypeptides. Importantly, the fragments remained associated within the tetramer and were only released upon activation, either in the gas phase or following shear stress. Crucially, fibril formation is then triggered only by the release of the fragments.

Molecular dynamics simulations suggest that after proteolysis, the tetramer experiences a loss of secondary structure and becomes unstable. Our mass spectrometry results show that release of the cleaved 1–48 and 49–127 fragments, and of the full-length protomer, occurred sequentially with increasing activation energy. The experimental mass spectrometry observations are therefore completely consistent with our MD simulations both demonstrating that the 1–48 fragment is the first to be released, evidently since it participates in fewer subunit interactions.

The *in vivo* circumstances of TTR cleavage and subsequent fibril formation are of considerable interest. Amyloid deposits formed *in vivo* by the majority of TTR variants contain the truncated 49–127 polypeptide (Ihse *et al*, 2013), but the identity and location of the protease responsible for the pathogenic cleavage are not known. We show here that cleavage can remain hidden within fully assembled, native S52P TTR tetramers and it is possible that proteolytic cleavage and tetramer disassembly may occur *in vivo* in different tissues and/or in different compartments of the same tissue.

Although some amyloidogenic TTR variants were apparently resistant to proteolysis (Mangione *et al*, 2014), we have shown here that susceptibility to proteolytic cleavage is enhanced by increasing shear stress. The amyloidogenic variants L55P, V122I, V30M and wild-type TTR, which itself is amyloidogenic in senile TTR amyloidosis, become susceptible to proteolytic cleavage and disassembly of the truncated protomer under increasing shear stress. Only the non-pathogenic T119M TTR variant, expression of which actually protects carriers of amyloidogenic mutations from developing amyloidosis (Hammarstrom *et al*, 2003), is resistant to proteolysis and fibrillogenesis. Based on our previous experiments (Mangione *et al*, 2013), we calculated that the shear and hydrophobic forces acting on TTR molecules under our fibrillogenesis conditions range from 5 to 100 pN. Such force is most likely sufficient to perturb the structure of the native protein (Cecconi *et al*, 2005; Borgia *et al*, 2008) exposing the C-D loop for proteolytic cleavage in all TTR variants examined with the exception of T119M.

The physiological relevance of protein activation by biomechanical forces is well established in the metabolism of von Willebrand factor (Zhang *et al*, 2009), a multimeric assembly in which cleavage of the A2 domain by the metalloprotease ADAMTS13 occurs only under fluid shear stress. In the case of TTR, it is not known whether the mechano-enzymatic processing of the protein affects normal catabolism of the protein or only contributes to amyloid formation. In the latter case, clinical and biochemical observations suggest that the genesis of the truncated peptide and its deposition could determine the natural history and prognosis (Gustafsson *et al*, 2012) of the disease. Indeed, we propose here that proteolytic cleavage enabled by sufficient biomechanical forces may importantly influence the tissue specificity of TTR amyloid deposition. Shear and interfacial forces generated by fluid flow (Stoppini & Bellotti, 2015) are particularly high in certain organs, especially in the heart where TTR amyloid is commonly deposited. The contribution of hydrophobic forces generated by extracellular ubiquitous macromolecules, which are crucially involved in the amyloid deposition (Bellotti & Chiti, 2008), is unclear. Data on the intensity of shear stress in the heart *in vivo* are difficult to obtain; however, Dokos and collaborators show that the shear forces generated in conditions mimicking those present in the ventricular myocardium are similar or even stronger (Dokos *et al*, 2000, 2002) to those used in our experimental conditions. Squeeze flow, as occurs in the heart, could further contribute to protein deformation, as observed with various types of polymers (Rowland *et al*, 2008). Also, TTR variants differ in stability and susceptibility to proteolytic cleavage. Thus, S52P TTR is readily cleaved under static conditions but requires shear stress forces for dissociation of the cleaved protomers, while wild-type TTR is cleaved only when exposed to stronger shear forces than those sufficient to dissociate the truncated subunit(s) from the tetramer.

**Figure 7:**
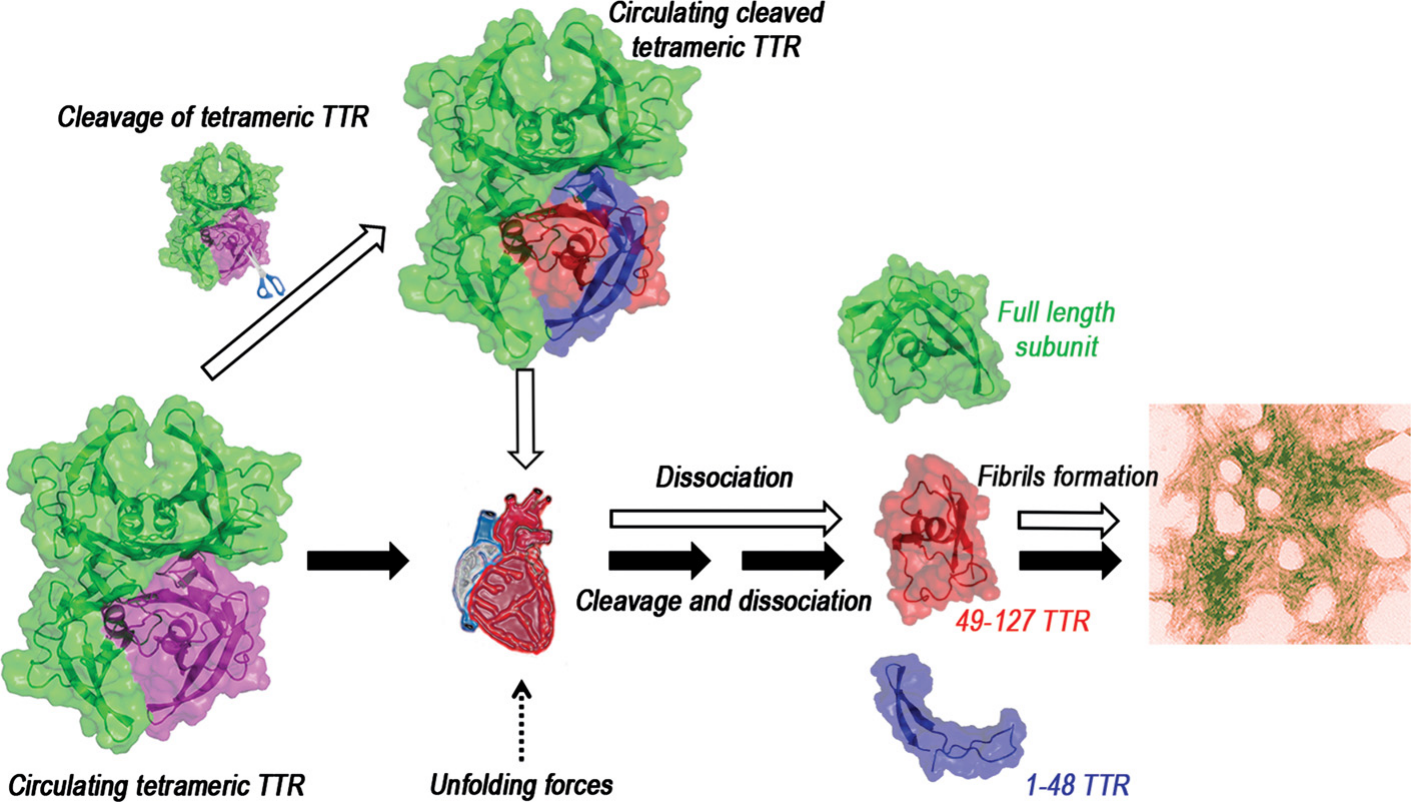
Amyloidogenesis of TTR primed by limited proteolysis *in vivo* Scheme of the sequential events which, *in vivo,* may occur after specific cleavage of TTR, as exemplified by of the S52P variant, with release of the truncated species and full-length protomers (open arrows) in the heart where shear and interfacial forces are the highest. Alternatively, the forces required to cleave other amyloidogenic variants including wild-type TTR (solid arrows) may enable the release of both the 49–127 fragment and the full-length protomer which can then aggregate into fibrils.

These findings combine to provide a compelling mechanism that is consistent with the propensity for TTR amyloid deposition in the heart (Fig 7). In this model, TTR variants that are very sensitive to selective proteolytic cleavage, such as S52P TTR, circulate as tetramers, even if the 48–49 peptide bond is cleaved in one protomer. We hypothesize that, during transit through the heart, the strong local shear and interfacial forces cause release of the cleaved 49–127 peptide which is then rapidly incorporated into amyloid fibrils. Alternatively, cleavage and dissociation could occur simultaneously, in the same anatomical site, in which both strong shear stress and the relevant proteolytic enzyme are present (Fig 7). The mechano-enzymatic mechanism that we have identified here for TTR may also contribute significantly to amyloid formation by other proteins in which proteolytic cleavage enabled by shear stress *in vivo* could promote fibrillogenesis, since highly amyloidogenic truncated forms of the soluble precursors have been found in amyloid fibrils derived from monoclonal immunoglobulin light chains, serum amyloid A protein, β2-microglobulin and apolipoprotein AI (Merlini & Bellotti, 2003). The mechanism of proteolysis-mediated TTR amyloidogenesis will be validated in transgenic mice expressing S52P TTR variant. To reproduce experimentally the *in vivo* conditions, a better understanding of the shear stress occurring at the interstitial space in the human heart will also be crucial.

The new pathway of TTR amyloidogenesis, which we have now characterized *in vitro,* may have important implications for drug development, including the tetramer stabilizing drugs currently in clinical trials. These agents were designed or selected to inhibit only one step in the fibrillogenesis pathway, as the key role of proteolysis has not previously been recognized and inhibitory effects thereon have not yet been considered (Connelly *et al*, 2010). For example, ligand-mediated inhibition of the mechano-enzymatic pathway of amyloidogenesis may be different for each variant. We have preliminary data showing that tafamidis may inhibit V30M TTR fibrillogenesis (Appendix Fig S5) at a lower dose (IC_50_ = 6 μM ± 1.4) than the one required for V122I TTR (9 μM ± 0.7, Fig 6). Elucidation of the new pathway should also refocus drug discovery efforts towards molecules able to inhibit fibrillogenesis by inducing stabilization and protection of the preferential proteolytic sites. Furthermore, simultaneous occupancy of the two thyroxine binding pockets, such as the one achieved by the prototypic palindromic ligand, mds84 (Kolstoe *et al*, 2010), would overcome the problem of negative cooperativity of binding of the existing drugs and should improve protection against amyloidogenic misfolding of TTR.

## Materials and Methods

### Preparation of recombinant proteins

Recombinant wild-type and S52P TTR were expressed and purified as previously described (Lashuel *et al*, 1998). Isotopically double-labelled proteins were expressed using Spectra 9 minimal medium (^13^C, 98%; and ^15^N, 98%) (Cambridge Isotope Laboratories, Inc.). Hybrid wild-type/S52P TTR was prepared with equimolar mixtures of 9 μM homotetramer wild-type and S52P TTR left at 4°C for 72 h allowing the subunit exchange to reach the equilibrium (Keetch *et al*, 2005). All chemicals were purchased from Sigma (Poole, Dorset, UK) unless otherwise stated.

### Size-exclusion chromatography

Size-exclusion chromatography of S52P TTR after digestion with trypsin (5 ng/μl) (Promega Trypsin Gold Mass Spectrometry Grade) was performed using a Superdex 200 column on the ÄKTA Explorer apparatus (GE Healthcare). The column was equilibrated and eluted at 0.5 ml/min with phosphate-buffered saline, pH 7.4 (PBS). The eluted peak was analysed by SDS–homogeneous 15% PAGE (GE Healthcare) under reducing conditions and mass spectrometry analysis after sample denaturation with 25% acetonitrile and 0.05% formic acid.

### Native mass spectrometry

After limited proteolysis, TTR aliquots were buffer-exchanged with 200 mM ammonium acetate pH 7.4 using Micro Bio-Spin size-exclusion chromatography columns (Bio-Rad) and loaded into in-house prepared glass capillaries for nano-electrospray (Hernandez & Robinson, 2007).

Energy ramping experiments were performed on a Q-TOFII modified for the transmission of large complexes (Sobott *et al*, 2002) and a synaptG2 (Waters). Parameters were as follows: capillary, cone and transfer voltages were set to 1.3 kV, 80 and 5 V, respectively. The acceleration voltage in the collision cell was ramped from 20 to 70 V with 2 to 10 V increments. The backing pressure in the transfer region was set to 5 mbar. To follow kinetics of subunit exchange, light and heavy proteins were first buffer-exchanged and then equal amounts were incubated for 3 days. Aliquots were regularly analysed on a TOF instrument (LCT; Waters) with the following parameters: 1.1 kV and 50 V of the capillary and sample cone voltages, respectively, and 5 mbar of backing pressure. To investigate the dissociation of cleaved S52P TTR after complete subunit exchange with the wild-type isoform, equimolar mixtures of 50 μM light wild-type and heavy S52P TTR were incubated in PBS for 3 days at 4°C and then overnight with trypsin at 37°C. The resulting sample was then buffer-exchanged, and analysed on a Q-TOFII (Waters) with the same parameters as described above, except for the acceleration voltage in the collision cell set to 40 V for an optimal visualization of the fragments. Calibration was performed using caesium iodide at 100 mg/ml, and mass spectra were analysed with MassLynx V4.1.

### Molecular dynamics simulations

Three simulations were prepared: native tetrameric wild-type TTR and both native and Lys48-cleaved S52P variant TTR. As residue 48 is located far from the two different dimer interfaces, the four subunits were considered as independent and cleavage was performed on each protomer, in order to increase sampling. Available crystal structures only include a dimer (Mangione *et al*, 2014), and inclusion of symmetry mates within the crystal led to the creation of a tetrameric arrangement. Cleavage of all four TTR monomers in S52P tetramer was reproduced by inserting COO^−^ and NH_3_^+^ termini between residues Lys48 and Thr49. Based on their neighbourhood, in all systems histidine amino acids were ε-protonated. All systems were solvated in a box of TIP3P water, and neutralized with the addition of 0.15 M Na^+^ and Cl^−^ ions. Final systems had a size of approximately 60,000 atoms. Molecular dynamics simulations were performed using the Amber99SB (Hornak *et al*, 2006) force field on NAMD2.9 molecular dynamics engine (Phillips *et al*, 2005) with SHAKE algorithm constraining heavy-atom distances, and PME treating the electrostatic interactions in periodic boundary conditions. All the systems were first minimized with 2,000 conjugate gradient steps, and subsequently simulated using a 2 fs timestep. Systems were first equilibrated in the nPT ensemble for 0.5 ns, with a 10 kcal/mol constrain on protein alpha carbons. 300 K and 1 Atm were imposed by Langevin dynamics, using a damping constant of 1/ps, a piston period of 200 fs and a piston decay of 50 fs. Constraints were subsequently removed, and systems were simulated in the nVT ensemble at 300 K for 1 ns. Finally, production runs for wild-type, S52P and S52P cleaved systems were run in the nPT ensemble (300 K and 1 Atm) for 522, 523 and 679 ns, respectively. From every production simulation, one structure per nanosecond was finally extracted and its secondary structure content assigned using STRIDE as implemented in VMD’s timeline tool (Humphrey *et al*, 1996). The percentage of conserved secondary structure elements in each subunit was computed by comparing any frame to the first one. Movie and rendering of protein structures have been produced with VMD.

### Fibrillogenesis of hybrid tetramers

Samples of 100 μl recombinant wild-type/S52P TTR before and after the completion of subunit exchange, prepared as described above, were incubated in PBS pH 7.4 containing 10 μM thioflavin T (ThT) (Mangione *et al*, 2014) at 37°C in Costar 96-well black-wall plates sealed with sealing films. Homotetramer wild-type and S52P TTR at 9 μM, respectively, were analysed in the same plate and subjected to 900-rpm double-orbital shaking (BMG LABTECH FLUOstar Omega), and bottom fluorescence was recorded as previously described (Mangione *et al*, 2014). After 1 h, 5 ng/μl trypsin or buffer alone was added and fluorescence was monitored in three or more replicates and control wells for the next 5 h. Fibrillar material was analysed by SDS–homogeneous 15% PAGE under reducing conditions. The effect of both tafamidis (Bulawa *et al*, 2012) and diflunisal (Berk *et al*, 2013) on fibrillogenesis of homotetramer S52P TTR (18 μM) was explored after incubating each protein sample at 37°C for 30 min in the presence of ligands at one-, two-, five-, eight- and tenfold molar excess, respectively. Protein/ligand complexes were then placed into 96-well black-wall plates and the fibrillogenesis procedure was carried out as described above. After the addition of trypsin to each well, ThT fluorescence was monitored for at least 10 h. The data were normalized to the signal plateau at 10 h after the initiation of each reaction.

### Cleavage and fibrillogenesis of other amyloidogenic TTR variants and wild-type TTR

Fibrillogenesis of L55P, V122I, V30M, and wild-type alongside S52P and the non-amyloidogenic T119M TTR variant was carried out in glass vials stirred at 1,500 rpm (IKA magnetic stirrer) and 37°C using 18 μM TTR in PBS pH 7.4 in the presence and in the absence of trypsin (5 ng/μl). The glass vial had an air/water interface of 1.5 cm^2^. Turbidity at 400 nm was monitored over time until it reached a plateau (72 h). Samples were analysed by SDS–homogeneous 15% PAGE (GE Healthcare) under reducing conditions, and bands corresponding to the intact TTR protomer were quantified with Quantity One software (Bio-Rad). After staining with alcoholic acid Congo red (CR), aggregated material was observed with polarized light microscopy. Morphology of wild-type TTR aggregates was characterized by electron microscopy as previously described (Mangione *et al*, 2014). Fibrillogenesis of V122I TTR was monitored by light scattering at 400 nm in the presence and the absence of 0.5-, one-, two- and fourfold molar excess of tafamidis (Bulawa *et al*, 2012), diflunisal (Berk *et al*, 2013) and mds84 (Kolstoe *et al*, 2010). The effect of tafamidis on V30M TTR fibril formation was also monitored as described above. For all the ligand inhibition experiments, the time of incubation of TTR with trypsin at 37°C was extended to 96 h. The ThT assay could not be used because mds84 strongly interferes with the fluorescence measurement. Quantification of the band of the full-length protomers in SDS–PAGE was performed as measurement of the susceptibility to proteolysis of TTR as described above. Experimental data were fitted to nonlinear regression curves using GraphPad Prism v5. Concentrations of ligand-inhibiting TTR fibril formation by 50% (IC_50_) were determined from aggregation curves and expressed as mean ± SD of three independent experiments.

### Role of shear and interfacial forces in the cleavage of wild-type and other amyloidogenic TTR variants

Structural destabilization of wild-type and L55P, V30M and V122I TTR variants might enable proteolytic cleavage of the protein. We investigated the role that shear forces (Bekard *et al*, 2011) and hydrophobic interactions (Chandler, 2005) might play in this process under our experimental conditions. To estimate the shear forces acting on the TTR tetramers in our system, we applied the model described by Shankaran & Neelamegham (Shankaran & Neelamegham, 2004). According to this model, the shear force (*F*_s_) acting on a molecule can be calculated as:

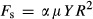

where α is the force coefficient, μ is the dynamic viscosity and γ is the shear rate (*dv*/*dx*). We considered TTR as a dumb-bell-shaped molecule with each end having a radius *R* of 3 nm. The value of α for this molecular geometry is 10 as previously reported (Shankaran & Neelamegham, 2004), where the shear rate for our system is 438 s. Using these parameters, we calculated a shear force *F*_*s*_ acting on TTR of ∼10^−17 ^N. This force is about 5 orders of magnitudes smaller than the forces required to destabilize the structure of a protein (Cecconi *et al*, 2005; Borgia *et al*, 2008), and thus, under our experimental conditions, *F*_*s*_ should not play an important role for TTR fibrillogenesis.

To estimate the hydrophobic interaction forces acting on the tetramer at the air/water interface (AWI), we calculated the hydrophobic interaction energy (*E*_*Hydro*_) as (Donaldson *et al*, 2011):

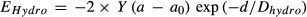

where *Υ* is the interfacial tension, *d* is the distance between the tetramer and the AWI, *a* is the exposed hydrophobicity of the molecule at distance *d*,*a*_*0*_ is the exposed hydrophobicity of one amino acid and *D*_*hydro*_ is the hydrophobic decay length. By taking the derivative of *E*_*Hydro*_ with respect to *d*, the hydrophobic force (*F*_*Hydro*_) acting on the molecule at AWI was calculated as:

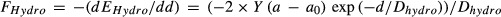

For TTR tetramer, we used *Υ* = 50 mJ/m^2^, as employed in a similar system (Johnson *et al*, 1971), *a*_0_ = 50 Å^2^, *D*_*hydro*_ = 10 Å (Israelachvili, 2011) and *a(d)* *= a*_*0*_ (*1 – exp*(*−d/D*_*hydro*_))^−1/2^. Considering the above parameters, we estimated that within 10 Å from the AWI, the hydrophobic forces (*F*_*Hydro*_) acting on the molecule vary from 5 to 100 pN.

## Supporting Information

Appendix

Movie EV1

Review Process File
