# Supplementary material for: A novel mechano-enzymatic cleavage mechanism underlies transthyretin amyloidogenesis
Source: EMBO Mol Med. 2015 Aug 18;7(10):1337–49. doi: 10.15252/emmm.201505357 (PMC4604687; doi:10.15252/emmm.201505357)
Supplement: Supplementary file 1 [file emmm0007-1337-sd1.pdf]

Appendix

A novel mechano-enzymatic cleavage mechanism underlies transthyretin amyloidogenesis

Julien Marcoux, P. Patrizia Mangione, Riccardo Porcari, Matteo T. Degiacomi, Guglielmo Verona, Graham W. Taylor, Sofia Giorgetti, Sara Raimondi, Sarah Sanglier-Cianférani, Justin L.P. Benesch, Ciro Cecconi, Mohsin M. Naqvi, Julian D. Gillmore, Philip N. Hawkins, Monica Stoppini, Carol V. Robinson, Mark B. Pepys and Vittorio Bellotti

Table of contents

	Page
Appendix Figure S1	2
Appendix Figure S2	3
Appendix Figure S2	4
Appendix Figure S4	5
Appendix Figure S5	6
Appendix Figure S6	7-8
Appendix Figure S7	9-10

Appendix Figure S1

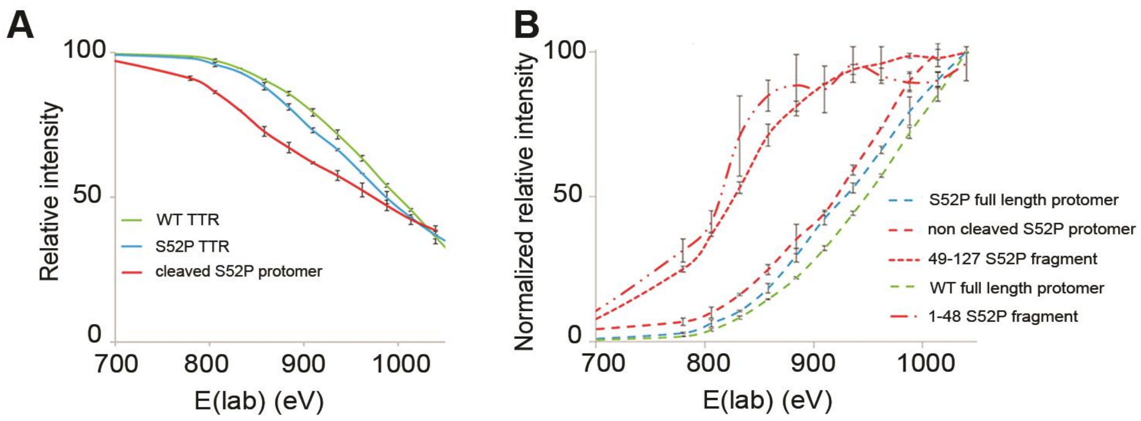

Appendix Fig S1. S52PTTR is destabilised after limited proteolysis. (A) Relative intensity of the tetramers at increasing energies. The wild type (green) is more stable than S52P TTR. After limited proteolysis (red), S52P TTR is even more unstable than the non-cleaved precursor (blue). (B) Both fragments are released at lower energy than the full length protomers. As expected, the non-cleaved fraction of the S52P TTR (long red dashes) shows the same profile as the protein solution before proteolysis (blue dashes). All the curves in red represent proteins present in the mixture after trypsin digestion. Data shown are mean ± SD of three replicates.

Appendix Figure S2

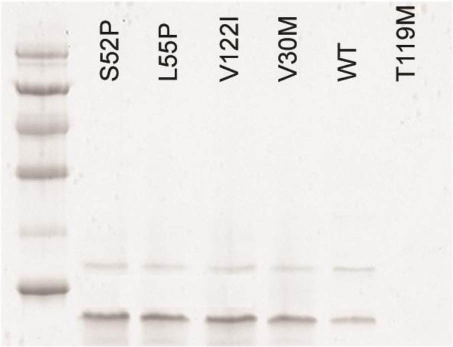

Appendix Fig S2. Composition of TTR fibrillar precipitate. SDS 15% PAGE under reducing conditions of fibrillar TTR aggregates present in the precipitate of trypsin-treated WT TTR and S52P, L55P, V122I and V30M variant TTR shown in Figure 5. The main component of fibrils is fragment 49-127. The stable variant, T119M TTR, does not form fibrils under these conditions. Marker proteins: 14.4, 20.1, 30.0, 45.0, 66.0, and 97.0 kDa.

Appendix Figure S3

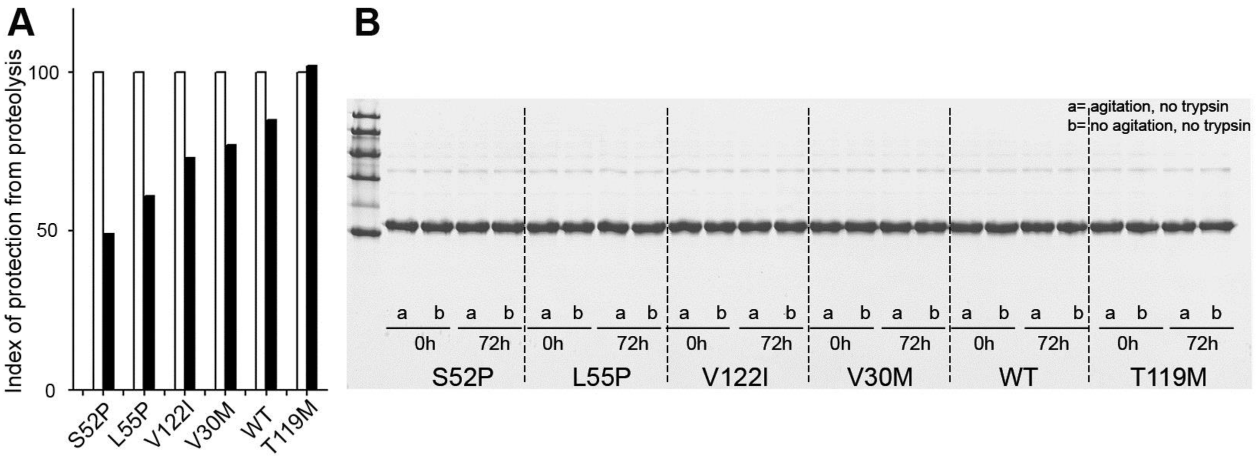

Appendix Fig S3. Sensitivity of TTR variants to proteolysis and effect of agitation alone. (A) The intensity of electrophoretic band corresponding to the intact TTR protomer remaining in the protein sample in the absence (open bars) and after 72 h incubation with trypsin (solid bars) as shown in Figure 5B. The level of undigested protomer is an index of the susceptibility to proteolysis of each TTR variant. (B) Under agitation and in the absence of trypsin (a), none of the TTR isoforms were cleaved after 72 h incubation, 37 °C as shown in the SDS 15% PAGE under reducing conditions. Control samples without both agitation and trypsin (b) were also included. Marker proteins: 14.4, 20.1, 30.0, 45.0, 66.0, and 97.0 kDa.

Appendix Figure S4

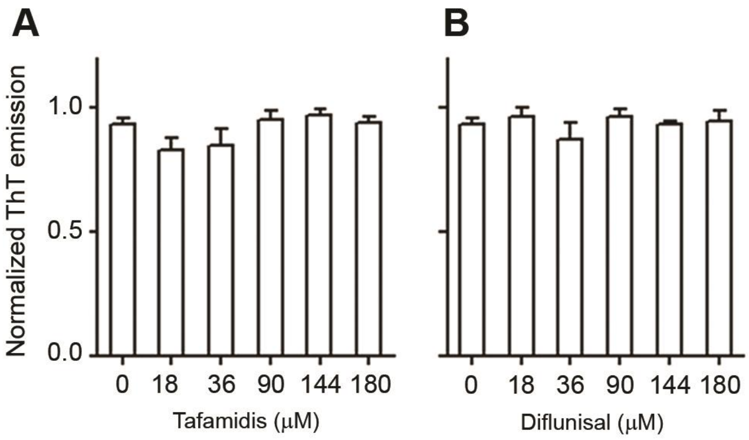

Appendix Fig S4. Lack of effect of TTR stabilizers on proteolysis/fibrillogenesis of S52P TTR. (A) Dose response of tafamidis and (B) diflunisal on fibrillogenesis by S52P TTR respectively. The data were normalized to the thioflavin T signal plateau at ~10h after the initiation of each reaction in the samples without drug. Means ± SD of three replicates are shown.

Appendix Figure S5

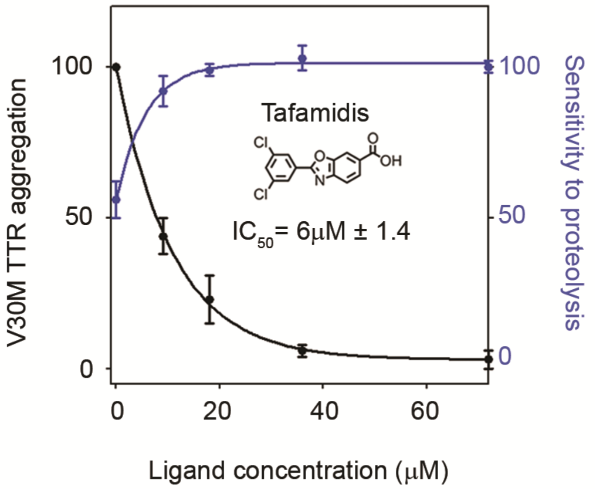

Appendix Fig S5. Proteolysis and fibrillogenesis of V30M TTR in the presence of tafamidis. Aggregation of 18 µM V30M TTR was monitored by turbidity at 400 nm after addition of trypsin in presence of 0, 9, 18, 36 and 72 µM of tafamidis for 96 h; aliquots of each sample were analysed by SDS 15% PAGE under reducing conditions. Values of turbidity (black line) at 400 nm were normalized to 100% for aggregation of the protein alone. Intensities of the SDS-PAGE band corresponding to the intact protomer in the whole mixture (blue line) were normalized to 100% for the same band of the protein before addition of trypsin. The solid lines represent the nonlinear fit to the experimental data using GraphPad Prism v5. IC_{50} value for TTR aggregation and tafamidis structure are included. All data shown represent mean ± SD of three independent experiments.

Appendix Figure S6

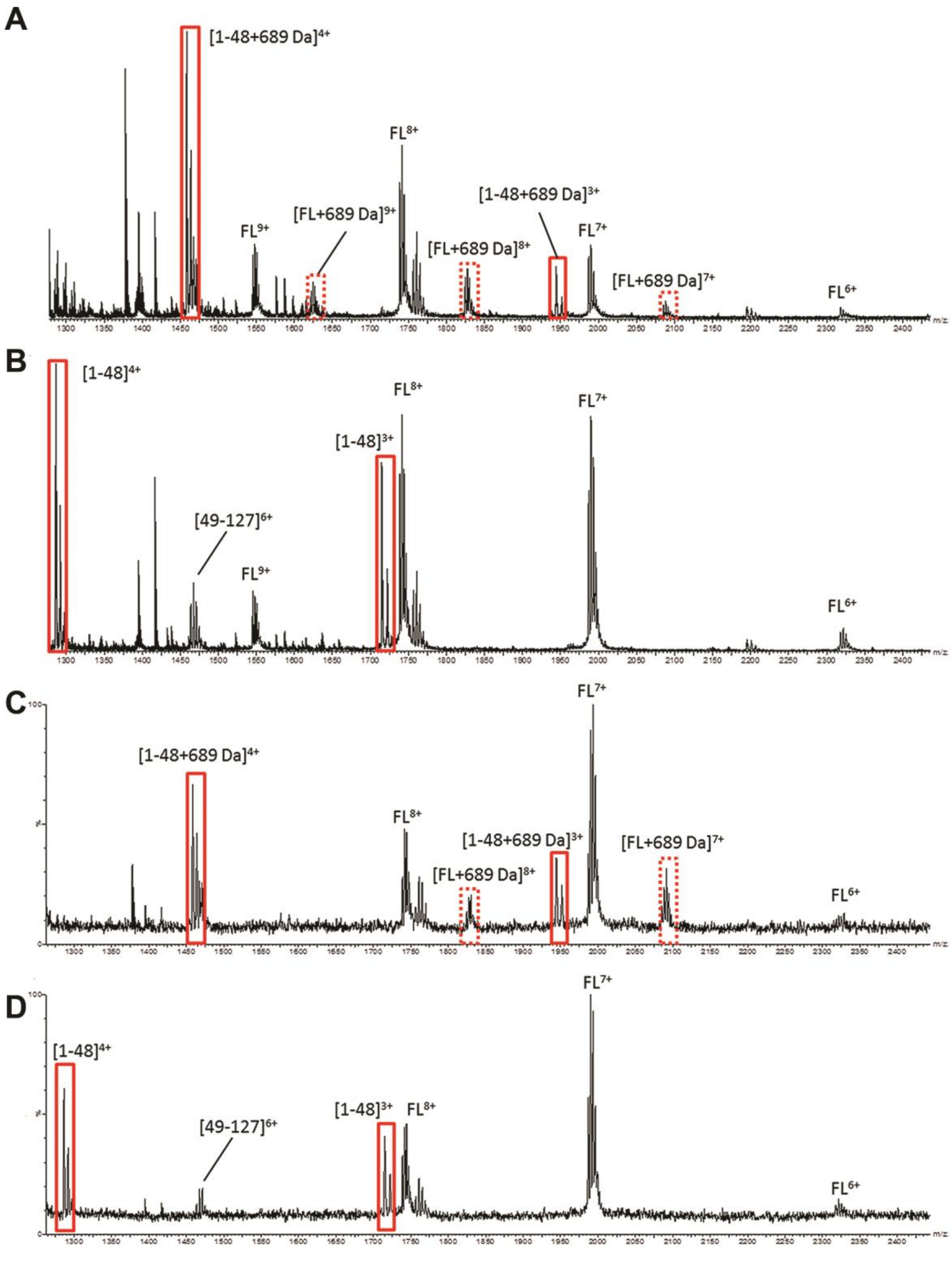

Appendix Fig S6. Observation of a post-proteolytic adduct. Native MS of recombinant S52P TTR at high collision energy, after overnight incubation with trypsin before (A) and after (B) incubation with 500 µM dithiothreitol. Upon addition of reducing agent, we observe a mass shift of 689 Da for both 1-48 and full length (FL) protomer components. The same mass shift was then observed after selection and activation of the tetrameric S52P TTR before (C) and after (D) addition of dithiothreitol, showing that this modification can occur at the tetramer level.

Appendix Figure S7

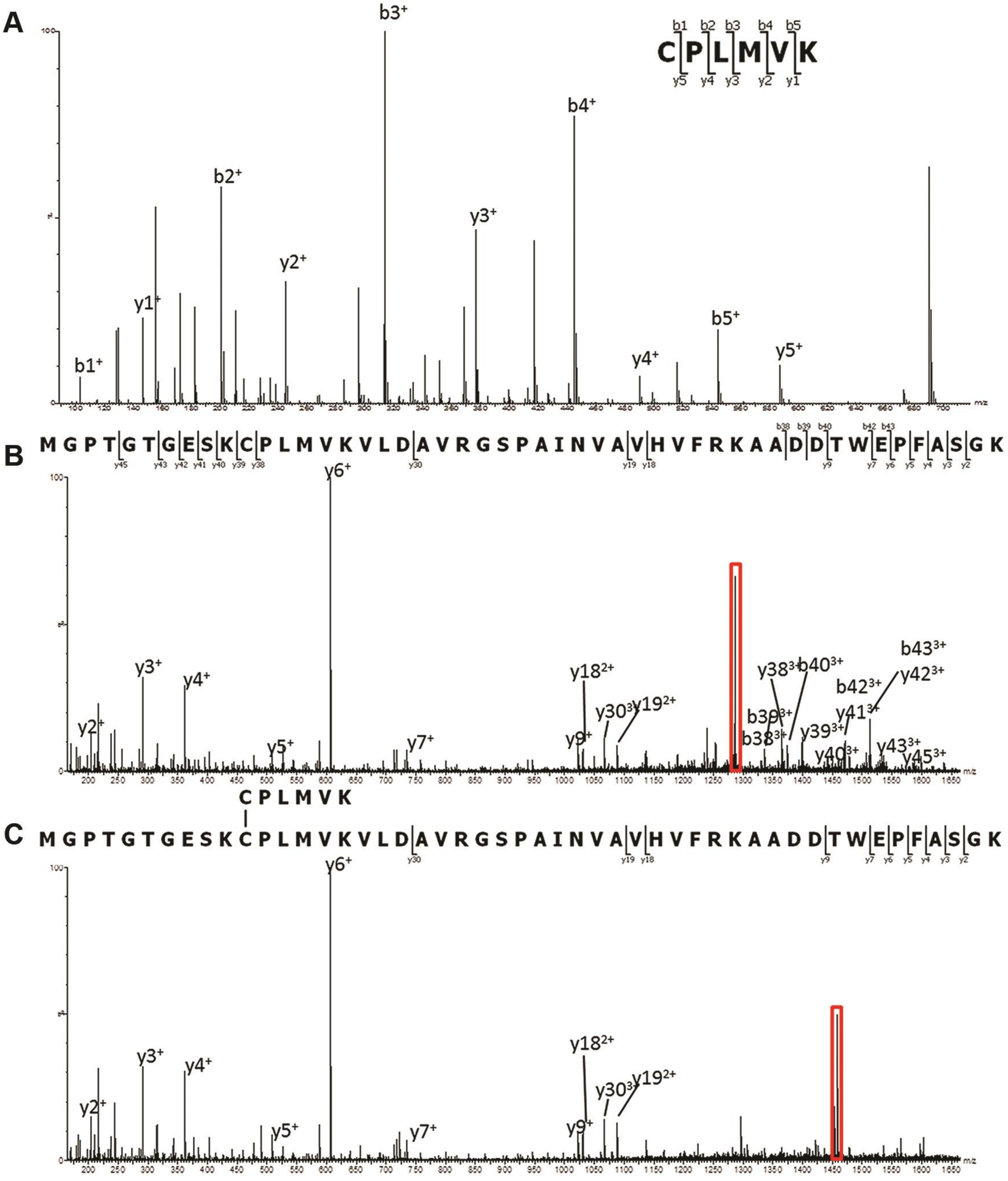

Appendix Fig S7. Identification of the post-proteolytic adduct. (A) After selection and MSMS fragmentation under reducing conditions (500 µM dithiothreitol), the ion of 689.4 Da was identified as the CPLMVK tryptic residue 11-16 fragment. The residue 1-48 fragment was also selected and fragmented by MSMS before (B) and after (C) reduction, yielding typical collisional induced dissociation fragments, as expected. The tryptic peptide 11-16 is thus able to bind to cysteine 11 of the full length protomer or of the 1-48 fragment.
