# Supplementary material for: A novel mechano-enzymatic cleavage mechanism underlies transthyretin amyloidogenesis
Source: EMBO Mol Med. 2015 Aug 18;7(10):1337–49. doi: 10.15252/emmm.201505357 (PMC4604687; doi:10.15252/emmm.201505357)
Supplement: Supplementary file 2 [file emmm0007-1337-sd2.zip › Movie_EV1_legend.rtf]

Movie EV1The video demonstrates that the selective cleavage at Lys48 in S52P TTR dramatically induces the unfolding of the otherwise stable residue 43-48 strand, accompanied by the allosteric loss of the EF-helix.
