# Supplementary material for: A novel mechano-enzymatic cleavage mechanism underlies transthyretin amyloidogenesis
Source: EMBO Mol Med. 2015 Aug 18;7(10):1337–49. doi: 10.15252/emmm.201505357 (PMC4604687; doi:10.15252/emmm.201505357)
Supplement: Supplementary file 3 [file emmm0007-1337-sd3.pdf]

EMBO Molecular Medicine

A novel mechano-enzymatic cleavage mechanism underlies transthyretin amyloidogenesis

Julien Marcoux, P. Patrizia Mangione, Riccardo Porcari, Matteo T. Degiacomi, Mr. Guglielmo Verona, Graham W. Taylor, Sofia Giorgetti, Sara Raimondi, Sarah Sanglier Cianférani, Justin L.P. Benesch, Ciro Cecconi, Moshin M. Naqvi, Julian D. Gillmore, Philip N. Hawkins, Monica Stoppini, Carol V. Robinson, Mark B. Pepys and Vittorio Bellotti

Corresponding author: Vittorio Bellotti, University College London

Review timeline:

	Submission date:	29 April 2015
	Accepted:	29 July 2015

Transaction Report:

No Peer Review Process File is available with this article, as the authors have chosen not to make the review process public in this case.

Editor: Céline Carret
